# Genetic polymorphism related to monocyte-macrophage function is associated with graft-versus-host disease

**DOI:** 10.1038/s41598-017-15915-3

**Published:** 2017-11-15

**Authors:** Kati Hyvärinen, Jarmo Ritari, Satu Koskela, Riitta Niittyvuopio, Anne Nihtinen, Liisa Volin, David Gallardo, Jukka Partanen

**Affiliations:** 1 Finnish Red Cross Blood Service, Helsinki, Finland; 2 Helsinki University Hospital, Comprehensive Cancer Center, Stem Cell Transplantation Unit, Helsinki, Finland; 3 Department of Hematology, Institut Català d’Oncologia, Hospital Dr. Josep Trueta, Girona, Spain

## Abstract

Despite detailed human leukocyte antigen (HLA) matching and modern immunosuppressive therapy, severe graft-versus-host disease (GvHD) remains a major hurdle for successful allogeneic hematopoietic stem cell transplantation (HSCT). As the genetic diversity in GvHD complicates the systematic discovery of associated variants across populations, we studied 122 GvHD-associated single nucleotide polymorphisms (SNPs) in 492 HLA-matched sibling HSCT donor-recipient pairs from Finland and Spain. The association between these candidate SNPs and grade III–IV acute GvHD and extensive chronic GvHD was assessed. The functional effects of the variants were determined using expression and cytokine quantitative trait loci (QTL) database analyses. Clear heterogeneity was observed in the associated markers between the two populations. Interestingly, the majority of markers, such as those annotated to IL1, IL23R, TLR9, TNF, and NOD2 genes, are related to the immunological response by monocytes-macrophages to microbes, a step that precedes GvHD as a result of intestinal lesions. Furthermore, cytokine QTL analysis showed that the GvHD-associated markers regulate IL1β, IFNγ, and IL6 responses. These results support a crucial role for the anti-microbial response in GvHD risk. Furthermore, despite apparent heterogeneity in the genetic markers associated with GvHD, it was possible to identify a biological pathway shared by most markers in both populations.

## Introduction

Allogeneic hematopoietic stem cell transplantation (HSCT) is a well-established curative treatment for many hematological malignancies. Graft-versus-host disease (GvHD) is the major life-threatening complication of HSCT. GvHD is mediated by donor immune cells in the graft, which recognize the patient’s tissues as foreign and destroy them. As the recipient is immunocompromised due to conditioning and immunosuppressive medication, the immune system of the recipient is not able to kill the foreign cells of the graft. GvHD occurs in 20–50% of HSCTs^1^.

One critical step in GvHD initiation is extensive immune activation due to microbial antigens that leak from the gastrointestinal track due to conditioning. The microbial antigens are detected by antigen-presenting cells (APCs) via pathogen-associated molecular pattern (PAMP) receptors, such as Toll-like receptors (TLRs) and NODs, leading to the activation of these cells. The activation of APCs leads to a cytokine storm, i.e., the production of high levels of cytokines, such as interleukin (IL)6, interferon (IFN)γ, IL23 and tumor necrosis factor (TNF) which, in turn, activate other immune cells^1–3^.

The outcome of HSCT is strongly influenced by the genetic differences between recipient/donor pairs^4^. The golden rule is genetic similarity or identity in the human leukocyte antigen (HLA) genes located in the major histocompatibility complex (MHC) on chromosome 6. However, there is evidence that HLA identity is not sufficient to prevent GvHD. Minor histocompatibility antigens^5^, mismatches in gene deletions^6^, non-HLA polymorphisms in immunoregulatory molecules^7–12^, drug-metabolizing genes^13,14^, and regulatory elements e.g., non-coding RNAs^11^, affect the risk of adverse outcomes^15^. In fact, we can assume GvHD as a multifactorial trait with a genetic component, in which the HLA matching is a crucial but not sufficient factor. As noted by Warren *et al*.^4^, while clinical HLA testing – sequencing of certain HLA gene exons - certainly focuses on functionally relevant variations, it encompasses only 1/1000^th^ of the entire MHC sequence, or 1000000^th^ part of the whole genome.

Genome-wide association (GWA) studies provide a systematic view of the genetic architecture and genetic risk markers of GvHD. One challenge for GWA studies in HSCT is diagnostic and treatment heterogeneity and the fact that the outcome may depend on properties of both the donor and recipient. It has been estimated that at least a few thousand HSCTs should be studied to reach sufficient statistical power for a GWA study^4,15^. To begin to tackle this challenge, we used an alternative approach described by Chien *et al*.^10^ and screened previously reported genetic associations in two additional populations with the rationale that, if replicated independently in many populations, the association may be genuine. We evaluated the previously reported GvHD-associated single nucleotide polymorphisms (SNPs) in two HLA-matched sibling allogeneic HSCT cohorts derived from Finnish and Spanish populations. To obtain coherent genetic information, the Immunochip array data were imputed and strictly filtered. We further analyzed the downstream functional effects of the associated SNPs to identify the disease-related biological pathways involved.

## Results

### Selection of the candidate SNPs

The 40 SNPs discovered by Chien *et al*.^10^ were included in the present analysis. The PubMed literature search from April 30, 2011 to January 31, 2017 identified 26 studies reporting an additional 82 SNPs associated with GvHD. From these 122 SNPs, 50 and 64 were found in the imputed and filtered Finnish and Spanish genotype datasets, respectively. The complete list of analyzed SNPs is presented in Supplementary Tables 1 and 2, and detailed data of imputed and genotyped SNPs associated with GvHD in the current study are depicted in Supplementary Tables 2 and 3. All Hardy–Weinberg equilibrium (HWE) P-values were >6 × 10^−4^ and no call rate was less than 93%. Metrics depicting the success of imputation were close to 1, implicating that these SNPs were imputed with high certainty.

### Candidate SNPs associated with acute and chronic GvHD in the Finnish cohort

In the Finnish cohort, we evaluated the association between 50 candidate SNPs and both acute GvHD (aGvHD) and chronic GvHD (cGvHD) outcomes. A summary of these associations at an α-level <0.05 is presented in Table 1.

**Table 1:** Candidate SNPs associated with acute and chronic GvHD in the Finnish cohort.

Outcome	Recipient/Donor	Gene*	SNP^†^	CHR	A1	A2	Odds ratio (95% confidence interval)^‡^	*P* ^‡^
aGvHD	Recipient	MICD	rs2523957	6	G	A	2.82 (1.51–5.27)	0.001
aGvHD	Recipient	PRSS53/VKRC1	rs7294	16	T	C	2.26 (1.21–4.25)	0.010
aGvHD	Donor	IL1R1	rs3917225	2	A	G	1.92 (1.09–3.37)	0.022
aGvHD	Donor	MICD	rs2523957	6	G	A	2.75 (1.55–4.87)	<0.001
aGvHD	Donor	TNF	rs1800629	6	A	G	2.24 (1.07–4.70)	0.029
aGvHD	Donor	NFKBIA	rs2233409	14	A	G	1.97 (1.05–3.70)	0.031
cGvHD	Recipient	IL1B	rs16944	2	A	G	1.55 (1.00–2.38)	0.047
cGvHD	Recipient	NOD2	rs6500328	16	G	A	1.58 (1.03–2.41)	0.035
cGvHD	Donor	HSPA1L	rs2075800	6	T	C	0.62 (0.38–0.99)	0.046
cGvHD	Donor	KRAS	rs1137282	12	G	A	0.46 (0.24–0.88)	0.017

SNP indicates single nucleotide polymorphism; CHR, chromosome; A1, minor allele; A2, major allele; aGvHD, acute graft-versus-host disease grade III–IV; and cGvHD, extensive chronic graft-versus-host disease.

*Annotation of SNP according to National Center for Biotechnology Information dbSNP database.

^†^Only SNPs showing an association at an α-level <0.05 are presented.

^‡^Odds ratios and P-values have been determined with PLINK 1.07 standard case/control association analysis, 1df chi-square allelic test.

Two recipient and four donor genotypes displayed an association with aGvHD. These results demonstrate an association between recipient rs2523957 (MICD) and rs7294 (PRSS53/VKRC1) and an increased risk of aGvHD (P = 0.001 and 0.010, respectively). Among the donors, SNPs rs3917225 (IL1R1, P = 0.022), rs2523957 (MICD, P < 0.001), rs1800629 (TNF, P = 0.029), and rs2233409 (NFKBIA, P = 0.031) predisposed recipients to an adverse outcome. It is important to note that the minor allele G at rs2523957 (pseudogene MICD) was associated with an increased risk of aGvHD in both the recipient (odds ratio [OR] 2.82, 95% confidence interval [CI]: 1.51–5.27, P = 0.001) and donor (OR 2.75, 95% CI: 1.55–4.87, P < 0.001) genotypes.

Two recipient and two donor genotypes showed associations with cGvHD. Recipient genotypes at rs16944 (IL1b) and rs6500328 (NOD2) resulted in a borderline increased risk of cGvHD (P = 0.047 and 0.035, respectively). The donor missense genotype at rs2075800 (HSPA1L) displayed a borderline protective association with cGvHD (OR 0.62, 95% CI: 0.38–0.99, P = 0.046). A synonymous codon at rs1137282 (KRAS) reduced the risk of cGvHD (OR 0.46, 95% CI: 0.24–0.88, P = 0.017).

### Candidate SNPs associated with acute and chronic GvHD in the Spanish cohort

A summary of the associations identified between candidate SNPs and aGvHD and cGvHD in the Spanish cohort is presented in Table 2. In total, 64 SNPs were evaluated. Table 2 shows the associations with an α-level <0.05. In the Spanish cohort, four recipient and six donor genotypes were associated with aGvHD. In both the recipient and donor genotypes, the minor allele A at rs2800230 (not in the gene) was associated with protection from aGvHD (recipient OR 0.57, 95% CI: 0.34–0.95, P = 0.031; donor OR 0.58, 95% CI: 0.35–0.95, P = 0.029). The IL1B SNP rs1143634 (P = 0.012) and the IL1A-annotated SNP rs1800587 (P = 0.031) conferred susceptibility to aGvHD in the recipient genotype. The minor allele G at rs2862833 (Fas cell surface death receptor, FAS) was associated with protection from aGvHD (OR 0.52, 95% CI: 0.31–0.87, P = 0.013).

**Table 2:** Candidate SNPs associated with acute and chronic GvHD in the Spanish cohort.

Outcome	Recipient/Donor	Gene*	SNP^†^	CHR	A1	A2	Odds ratio^‡^ (95% confidence interval)	*P* ^‡^
aGvHD	Recipient	Not in gene	rs2800230	1	A	G	0.57 (0.34–0.95)	0.031
aGvHD	Recipient	IL1A	rs1800587	2	A	G	1.75 (1.05–2.92)	0.031
aGvHD	Recipient	IL1B	rs1143634	2	A	G	2.00 (1.16–3.45)	0.012
aGvHD	Recipient	FAS	rs2862833	10	G	A	0.52 (0.31–0.87)	0.013
aGvHD	Donor	IL10	rs1800872	1	T	G	2.19 (1.33–3.63)	0.002
aGvHD	Donor	IL10	rs1800871	1	A	G	2.19 (1.33–3.63)	0.002
aGvHD	Donor	IL10	rs1800896	1	C	T	0.48 (0.29–0.80)	0.004
aGvHD	Donor	IL10RB	rs2834167	21	G	A	1.96 (1.17–3.28)	0.010
aGvHD	Donor	Not in gene	rs2800230	1	A	G	0.58 (0.35–0.95)	0.029
aGvHD	Donor	LOC105373109	rs10737416	1	A	C	1.73 (1.05–2.85)	0.030
cGvHD	Recipient	IL23R	rs11209026	1	A	G	2.61 (1.13–6.04)	0.020
cGvHD	Donor	TLR9	rs352140	3	C	T	0.58 (0.37–0.91)	0.018
cGvHD	Donor	TLR9	rs352139	3	T	C	0.59 (0.38–0.93)	0.023

SNP indicates single nucleotide polymorphism; CHR, chromosome; A1, minor allele; A2, major allele; aGvHD, acute graft-versus-host disease grade III–IV; and cGvHD, extensive chronic graft-versus-host disease.

*Annotation of SNP according to National Center for Biotechnology Information dbSNP database.

^†^Only SNPs showing an association at an α-level <0.05 are presented.

^‡^Odds ratios and P-values have been determined with PLINK 1.07 standard case/control association analysis, 1df chi-square allelic test.

In Spanish donors, IL10 promoter SNPs rs1800872 and rs1800871 increased the risk of aGvHD (OR 2.19, 95% CI: 1.33–3.63, P = 0.002 for both SNPs). The IL10RB missense variation at rs11209026 was also associated with an increased risk of aGvHD (OR 1.96, 95% CI: 1.17–3.28, P = 0.010). However, the IL10 promoter SNP rs1800896 displayed a protective minor allele C association with the disease (OR 0.48, 95% CI: 0.29–0.80, P = 0.004).

Two donor genotype TLR9 SNPs (rs352140, P = 0.018 and rs352139, P = 0.023) were associated with protection from cGvHD. An adverse association with cGvHD was found in cases with the recipient genotype IL23R missense SNP rs11209026 (OR 2.61, 95% CI: 1.13–6.04, P = 0.020).

### Expression quantitative trait loci analysis of candidate SNPs

The downstream effects of GvHD-associated SNPs on mRNA expression were determined using the Blood expression quantitative trait loci (eQTL) Database generated by Westra *et al*.^16^. When analyzing all 21 SNPs showing an association with aGvHD or cGvHD in the current study, several SNPs were determined to affect the expression of nearby genes (Tables 3 and 4). No significant *trans*-eQTL effects were detected.

**Table 3:** eQTL analysis of candidate SNPs in the Finnish cohort.

Outcome	Recipient/Donor	Gene*	SNP	CHR	A1	Risk/protective^†^	*cis*-eQTL gene^‡^	Z-score^‡^	*P* ^‡^	FDR^‡^
aGvHD	Recipient + donor	MICD	rs2523957	6	G	Risk	HLA-F	15.64	3.65 × 10^–55^	<0.01
							HLA-G	−13.89	7.18 × 10^−44^	<0.01
							PPP1R11	−9.09	1.03 × 10−^19^	<0.01
							ZNRD1	5.44	5.27 × 10^−8^	<0.01
aGvHD	Recipient	PRSS53/VKRC1	rs7294	16	T	Risk	STX4	16.17	8.85 × 10^−59^	<0.01
							BCKDK	−11.27	1.82 × 10^−29^	<0.01
							ZNF668	−10.78	4.12 × 10^−27^	<0.01
							AC093520.4, ITGAM	−6.26	3.73 × 10^−10^	<0.01
							AC135050.5	−5.95	2.68 × 10^−9^	<0.01
							MYST1	5.58	2.38 × 10^−8^	<0.01
aGvHD	Donor	IL1R1	rs3917225	2	A	Risk	IL1R2	3.36	7.73 × 10^−4^	0.20
aGvHD	Donor	TNF	rs1800629	6	A	Risk	TNF	−5.28	1.28 × 10^−7^	<0.01
							CSNK2B	5.17	2.29 × 10^−7^	<0.01
							LTA	−3.21	1.31 × 10^−3^	0.29
aGvHD	Donor	NFKBIA	rs2233409	14	A	Risk	—	—	—	—
cGvHD	Recipient	IL1B	rs16944	2	A	Risk	NT5DC4	3.2	1.36 × 10^−3^	0.30
cGvHD	Recipient	NOD2	rs6500328	16	G	Risk	NOD2	−22.76	1.10 × 10^−114^	<0.01
cGvHD	Donor	HSPA1L	rs2075800	6	T	Protective	CSNK2B	−22.03	1.47 × 10^−107^	<0.01
							HSPA1B	20.06	1.78 × 10^−89^	<0.01
							HSPA1L	8.17	3.02 × 10^−16^	<0.01
							LY6G5C	5.64	1.75 × 10^−8^	<0.01
							RDBP	5.62	1.90 × 10^−8^	<0.01
							AIF1	5.35	8.77 × 10^−8^	<0.01
							BAT3	4.52	6.13 × 10^−6^	<0.01
cGvHD	Donor	KRAS	rs1137282	12	G	Protective	—	—	—	—

SNP indicates single nucleotide polymorphism; CHR, chromosome; A1, minor allele; eQTL indicates expressive quantitative trait loci; FDR, false detection rate; aGvHD, acute graft-versus-host disease grade III–IV; −, no *cis*-eQTL records found; and cGvHD, extensive chronic graft-versus-host disease.

*Annotation of SNP according to National Center for Biotechnology Information dbSNP database.

^†^Risk/protective outcome status of the SNP has been determined from the association results (Table 1).

^‡^Westra *et al.* ^16^. Blood eQTL Browser http://genenetwork.nl/bloodeqtlbrowser/.

**Table 4:** eQTL analysis of candidate SNPs in the Spanish cohort.

Outcome	Recipient/Donor	Gene*	SNP	CHR	A1	Risk/protective^†^	*cis*-eQTL gene^‡^	Z-score^‡^	*P* ^‡^	FDR^‡^
aGvHD	Recipient + donor	Not in gene	rs2800230	1	A	Protective	—	—	—	—
aGvHD	Recipient	IL1A	rs1800587	2	A	Risk	SLC20A1	−4.03	5.62 × 10^−5^	0.02
							CHCHD5	−3.59	3.31 × 10^−4^	0.11
aGvHD	Recipient	IL1B	rs1143634	2	A	Risk	CHCHD5	−4.1	4.10 × 10^−5^	0.02
							SLC20A1	−3.19	1.43 × 10^−3^	0.31
aGvHD	Recipient	FAS	rs2862833	10	G	Protective	STAMBPL1, ACTA2	38.25	9.81 × 10^−198^	<0.01
							FAS	10.8	3.51 × 10^−27^	<0.01
aGvHD	Donor	IL10	rs1800872	1	T	Risk	—	—	—	—
aGvHD	Donor	IL10	rs1800871	1	A	Risk	—	—	—	—
aGvHD	Donor	IL10	rs1800896	1	C	Protective	RASSF5	−3.51	4.56 × 10^−4^	0.14
aGvHD	Donor	LOC105373109	rs10737416	1	A	Risk	—	—	—	—
aGvHD	Donor	IL10RB	rs2834167	21	G	Risk	IL10RB	7.01	2.44 × 10^−12^	<0.01
							IFNAR1	6.14	8.10 × 10^−10^	<0.01
cGvHD	Recipient	IL23R	rs11209026	1	A	Risk	—	—	—	—
cGvHD	Donor	TLR9	rs352140	3	C	Protective	PPM1M	17.02	5.90 × 10^−65^	<0.01
							DNAH1	4.59	4.51 × 10^−6^	<0.01
cGvHD	Donor	TLR9	rs352139	3	T	Protective	PPM1M	17.2	2.71 × 10^−66^	<0.01
							DNAH1	7.31	2.69 × 10^−13^	<0.01

SNP indicates single nucleotide polymorphism; CHR, chromosome; A1, minor allele; eQTL indicates expressive quantitative trait loci; FDR, false detection rate; aGvHD, acute graft-versus-host disease grade III–IV; –, no *cis*-eQTL records found; and cGvHD, extensive chronic graft-versus-host disease.

*Annotation of SNP according to National Center for Biotechnology Information dbSNP database.

^†^Risk/protective outcome status of the SNP has been determined from the association results (Table 2).

^‡^Westra *et al.* ^16^. Blood eQTL Browser http://genenetwork.nl/bloodeqtlbrowser/.

In the Finnish cohort (Table 3), SNP rs2075800, located within the heat shock protein A1-like (HSPA1L) gene, protected individuals from cGvHD and was associated with increased expression of HSPA and the adjacent HSPA1B gene, with an FDR level <0.05 (HSPA1B Z-score 20.06, P = 1.78 × 10^−89^; HSPA1L Z-score 8.17, P = 3.02 × 10^−16^). In both the recipient and donor genotypes, aGvHD-predisposing rs2523957 (pseudogene MICD) was associated with reduced expression of HLA-G, with a Z-score = −13.89 and a P = 7.18 × 10^−44^, and increased expression of HLA-F, with a Z-score = 15.64 and a P = 3.65 × 10^−55^. Additionally, the *cis*-eQTL results for TNF revealed that the disease-predisposing SNP rs1800629 reduced expression of the TNF gene (P = 1.28 × 10^−7^). It is also important to note that the HSPA1L, HSPA1B, MICD, and TNF genes are all located in the MHC region. The intronic NOD2 rs6500328 decreased expression of the NOD2 gene (Z-score −22.76, P = 1.10 × 10^−114^) and the minor allele G was identified as a risk factor in the outcome association analysis (Table 1).

In the Spanish cohort, two TLR9-annotated SNPs were associated with GvHD (Table 4). The aGvHD-protective SNPs, rs352140 and rs352139, increased the expression of PPM1M (rs352140 Z-score 17.02, P = 5.90 × 10^−65^; rs352139 Z-score 17.2, P = 2.71 × 10^−66^). The protective SNP rs2862833, a downstream variant of the FAS gene, increased the expression of FAS (Z-score 10.8, P = 3.51 × 10^−27^) and the STAMBPL1/ACTA2 locus (Z-score 38.25, P = 9.81 × 10^−198^). IL10 promoter SNPs, rs1800872, rs1800871, and rs1800896, did not demonstrate any significant *cis*-eQTL association. The aGvHD-predisposing IL10RB missense SNP rs2834167 was associated with increased expression of IL10RB, with a Z-score = 7.01 and a P = 2.44 × 10^−12^.

### Cytokine QTL associations of candidate SNPs

The cytokine storm plays an important role in the initiation phase of GvHD. Therefore, we examined cytokine QTL effects of the associated SNPs by utilizing the cytokine QTL database recently published by Li Y *et al*.^17^. The results are presented in Tables 5 and 6.

**Table 5:** Cytokine QTL associations of candidate SNPs in the Finnish cohort.

Outcome	Recipient/Donor	Gene*	SNP	CHR	A1	Risk/protective^†^	Stimulus^‡^	Cell system^‡^	Stimulation days^‡^	Cytokine^‡^	*P* ^‡^
aGvHD	Recipient +donor	MICD	rs2523957	6	G	Risk	*Cryptococcus*	PBMC	1	IL6	0.009
aGvHD	Recipient	PRSS53/VKRC1	rs7294	16	T	Risk	*C*. *albicans* conidia	PBMC	1	IL6	0.015
aGvHD	Donor	IL1R1	rs3917225	2	A	Risk	*Bacteroides*	PBMC	7	INFy	0.014
aGvHD	Donor	TNF	rs1800629	6	A	Risk	*Cryptococcus*	PBMC	7	INFy	0.016
aGvHD	Donor	NFKBIA	rs2233409	14	A	Risk	—	—	—	—	—
cGvHD	Recipient	IL1B	rs16944	2	A	Risk	*S*. *aureus*	PBMC	7	INFy	0.003
cGvHD	Recipient	NOD2	rs6500328	16	G	Risk	*C*. *albicans* hyphae	PBMC	7	INFy	0.031
cGvHD	Donor	HSPA1L	rs2075800	6	T	Protective	PHA	WB	2	INFy	0.007
cGvHD	Donor	KRAS	rs1137282	12	G	Protective	—	—	—	—	—

SNP indicates single nucleotide polymorphism; CHR, chromosome; A1, minor allele; aGvHD, acute graft-versus-host disease grade III–IV; PBMC, peripheral blood mononuclear cell; − no cytokine quantitative trait loci records found; cGvHD, extensive chronic graft-versus-host disease; PHA, phytohaemagglutinin; and WB, whole blood.

*Annotation of SNP according to National Center for Biotechnology Information dbSNP database.

^†^Risk/protective outcome status of the SNP has been determined from the association results (Table 1).

^‡^The cytokine QTL database (https://hfgp.bbmri.nl/), published by Li Y *et al.* ^17^.

**Table 6:** Cytokine QTL associations of candidate SNPs in the Spanish cohort.

Outcome	Recipient/Donor	Gene*	SNP	CHR	A1	Risk/protective^†^	Stimulus^‡^	Cell system‡	Stimulation days^‡^	Cytokine^‡^	*P* ^‡^
aGvHD	Recipient + donor	Not in gene	rs2800230	1	A	Protective	Borreliamix	PBMC	7	INFy	0.045
aGvHD	Recipient	IL1A	rs1800587	2	A	Risk	*C*. *albicans* conidia	PBMC	7	INFy	0.003
aGvHD	Recipient	IL1B	rs1143634	2	A	Risk	*Cryptococcus*	PBMC	7	INFy	0.030
aGvHD	Recipient	FAS	rs2862833	10	G	Protective	*C*. *burnetii* Nine mile serum	PBMC	1	IL6	0.008
aGvHD	Donor	IL10	rs1800872	1	T	Risk	*E*. *Coli*	PBMC	1	IL1b	0.001
aGvHD	Donor	IL10	rs1800871	1	A	Risk	*E*. *Coli*	PBMC	1	IL1b	0.001
aGvHD	Donor	IL10	rs1800896	1	C	Protective	*B*. *burgdorferi*	PBMC	7	INFy	0.049
aGvHD	Donor	IL10RB	rs2834167	21	G	Risk	*C*. *albicans* hyphae	PBMC	7	INFy	0.034
aGvHD	Donor	LOC105373109	rs10737416	1	A	Risk	Borreliamix	PBMC	7	IL22	0.043
cGvHD	Recipient	IL23R	rs11209026	1	A	Risk	—	—	—	—	—
cGvHD	Donor	TLR9	rs352140	3	C	Protective	—	—	—	—	—
cGvHD	Donor	TLR9	rs352139	3	T	Protective	—	—	—	—	—

SNP indicates single nucleotide polymorphism; CHR, chromosome; A1, minor allele; aGvHD, acute graft-versus-host disease grade III–IV; PBMC, peripheral blood mononuclear cell; −, no cytokine quantitative trait loci records found; and cGvHD, extensive chronic graft-versus-host disease.

*Annotation of SNP according to National Center for Biotechnology Information dbSNP database.

^†^Risk/protective outcome status of the SNP has been determined from the association results (Table 2).

^‡^The cytokine QTL database (https://hfgp.bbmri.nl/), published by Li Y *et al*.^17^.

In the Finnish cohort, six of seven SNPs associated with an increased risk of GvHD were linked with alterations in the production of IL6 and IFNγ by peripheral blood mononuclear cells (PBMCs) at an alpha level <0.05 (Table 5). When examining the link between predisposing genotypes at MICD and PRSS53/VKRC1 loci and IL6, the initial stimuli were revealed as fungi *Cryptococcus* and *Candida albicans*. Changes in the production of IFNγ emerged following stimulation with *Bacteroides*, *Cryptococcus*, *Stafylococcus aureus*, and *C*. *albicans* combined with carrying a risk allele at IL1R1, TNF, IL1β, or NOD2, respectively. Donor genotype T at the HSPA1L-annotated SNP rs2075800 was combined with whole blood stimulus by phytohaemagglutinin and production of IFNγ (P = 0.007).

In the Spanish cohort, the majority of cytokine QTL associations focused on the IL1β and IFNγ responses of stimulated PBMCs (Table 6). Recipient SNPs at IL1α and IL1β aGvHD risk loci were significantly associated with altered production of IFNγ by PBMCs following stimulation with *C*. *albicans* (rs1800587 and rs1071676, P < 0.05 for both) or *Cryptococcus* (rs1143634, P = 0.030). Donor aGvHD-predisposing IL10 promoter region genotypes at rs1800872 and rs1800871 combined with the IL1β response of *Escherichia coli*-stimulated PBMCs (P = 0.001). However, the aGvHD-protective IL10 SNP, rs1800896, displayed a borderline association with the IFNγ response after stimulation with *Borrelia burgdorferi* (P = 0.049). Donor minor allele G at rs2834167 (IL10RB), having an adverse association with aGvHD, combined with altered production of IFNγ by *C*. *albicans*-stimulated PBMCs. The IL6 response was altered when *C*. *burnetii*-stimulated PBMCs were combined with the aGvHD-protective genotype at the FAS locus rs2862833 (P = 0.008).

## Discussion

To systematically identify genetic loci that are associated with GvHD, we screened previously reported SNPs for their genetic associations with aGvHD and cGvHD in a total of 492 HLA-matched sibling HSCT recipient-donor pairs. The cohorts were derived from two populations: Finnish and Spanish. The major finding of the present study was that, despite clear heterogeneity in GvHD-associated polymorphisms between the two populations, the markers share a common feature: they are predominantly annotated with genes that are important in the host response to microbial antigens. Furthermore, the functional effects of these polymorphisms were related to the same pathways.

The GvHD-associated genes included IL1, IL10, IL23R, TLR9, TNF, and NOD2, which all play a role in the host response to microbes. However, it was of further interest that the polymorphisms were determined to regulate the expression levels of cytokines IL1β, IL6, and IFNγ, all of which are important mediators of the cytokine storm.

Several SNPs annotated to pathogen recognition receptors (PRRs) have been previously associated with GvHD^7,8^. In TLR9, which detects intracellular bacterial single-stranded CpG-DNA, we found protective polymorphisms that showed no direct effect on expression of the TLR9 gene in two of the QTL databases utilized herein. The intronic risk SNP rs6500328 in the NOD2 gene was associated with reduced expression of the NOD2 gene and was also associated with IFNγ expression in the cytokine QTL database. The cytokine QTL analysis demonstrated complex crosstalk between the associated SNPs, their direct QTL effects and the response of particular cell populations to microbial antigens. To further support a role for PRRs in GvHD, our unpublished studies suggest that LPS-recognizing TLR4 displays intronic minor alleles at rs12377632 and rs1927907, both of which were associated with GvHD protection and a strong increase in TLR4 expression. The protective TLR4 genotype at rs1927907 was associated with the IL1β response of *C*. *burnetii*-stimulated PBMCs. However, their associations with aGvDH have not yet been reported; therefore, these SNPs were not included in the current study and should be further analyzed in other populations.

The use of eQTL and cytokine QTL databases allows for demonstration of the functional or downstream effects of disease-associated polymorphisms. The eQTL analyses performed herein revealed interesting findings and indicated shared pathways. The association between IL10 polymorphisms and GvHD has been established in many populations^18–21^ and it has been assumed to be related to different expression levels of IL10. However, the IL10 markers rs1800872 and rs1800871 determined to be associated in the present study showed no eQTL effects on IL10 expression, but rather the polymorphisms regulated the IFNγ and IL1β levels produced by PBMCs after stimulation with *E*. *coli* or *B*. *burgdorferi*. In contrast, missense polymorphisms in IL10RB did regulate the level of IL10RB and IFNγ in the cytokine eQTL. Hence, each polymorphism may exert various effects at different steps of the immune response. Unfortunately, the cytokine database in its present form shows no relationship between the allele and the direction of the measured cytokine response, making it difficult to interpret the mechanisms of risk alleles. In this regard, GvHD-specific QTL databases would facilitate the functional interpretation of significant variants.

A number of SNPs showing an association with GvHD risk in the Finnish cohort mapped to MHC on chromosome 6p21.3. TNF, MICD, and HSPA1L are located relatively close to each other; therefore, the observed associations may be derived from a single genetic polymorphism in linkage disequilibrium with the markers analyzed here. Alternatively, there may be multiple polymorphisms within the MHC segment associated with the disease. For example, emerging evidence indicates multiple novel MHC-associated risk markers for GvHD^4,22^. Based on the present results, it is not possible to pinpoint which marker is the primary or closest to the true risk polymorphism. In fact, as long as we do not know the causal SNP, these results only indicate that the genes annotated to SNP may influence the risk for GvHD. Differences in linkage disequilibrium between causal and studied SNPs result in discrepancies observed between results from different populations as seen also in the present study when compared to original findings.

In addition to ethnicity and the genotyping array performed, the two cohorts investigated in this study also differed from each other with respect to their clinical HSCT setting. The Finnish cohort was from a single center, whereas the Spanish cohort originated from a number of clinics. The stem cell source, conditioning regimen, and GvHD prevention procedures varied significantly and may have contributed to the heterogeneity of the GvHD-associated SNPs. Combining these typical aspects with well-established GvHD risk factors^2,3^, such as donor and recipient age, transplant gender direction, diagnosis and staging, and infections, may also partially explain why, despite numerous GvHD candidate genes and markers studied in recent years, the consistency of results across studies has been sparse.

To date, genome-wide studies in GvHD have been reported only among mostly Caucasian^23^ and Japanese^24^ populations. These studies have not reported overlapping or shared risk loci, indicating the heterogeneous nature of GvHD genetics. It will be of interest to test whether the utilization of eQTL or pathway approaches similar to those used in the present study would reveal common mechanisms behind apparently heterogeneous associations. While GWA studies investigating millions of variants require vigorous control of multiple tests, due to the replicative nature of the present study, an alpha value <0.05 was selected as the threshold for statistical significance for allele frequency association with GvHD. The fact that the associated genes were functionally relevant to GvHD can be regarded as additional supportive evidence.

As demonstrated by the present study and many other association studies^8–10,25^, individuals may carry genetic factors rendering them more susceptible to react immunologically against foreign structures, such as allogeneic cells or intestinal microbes. Such a high responder genotype may be helpful in clearing infections but may also increase the risk of GvHD. This may be one of the important genetic factors for GvHD risk. We can also assume that an increased risk of GvHD results from insufficient histocompatibility; despite good HLA matching, mismatches in minor histocompatibility antigens may also play a role in GvHD risk^5,6^. Another possibility is the pharmacogenomic differences in the response to or efficiency of the immunosuppressive treatment^13^. This has been scarcely explored but certainly merits further investigation. Hence, we constructed at least three overlapping models for GvHD genetics that most likely act together.

The present study provides further evidence that genetic variation regulating the level of the immune response against bacterial antigens is an important non-HLA factor in GvHD susceptibility. Although individual associated polymorphisms are not necessarily the same throughout different populations, they consistently belong to the same regulatory pathways participating in cytokine-mediated inflammation of the intestinal epithelium. It is likely that a similar type of heterogeneity can be found in other populations or cohorts, and it remains to be determined whether associated markers also belong to the same biological pathway. This heterogeneity implies that large genome screens may be needed for clinical GvHD predictions, rather than focusing on only a small number of selected genetic markers.

## Methods

### Literature search

Chien *et al*.^10^ identified 41 publications reporting 40 SNPs associated with aGvHD up to April 30, 2011, which were included in our analysis. We also performed a PubMed search using the term “acute GvHD AND polymorphism” to identify published studies reporting an association analysis of genetic variants with GvHD from April 30, 2011 until January 31, 2017. Studies reporting associations at an α-level >0.05 were excluded and, from all of the variant types, only SNPs were selected for further analysis.

### Study populations

The SNP association analyses were replicated within two separate populations. The characteristics of all recipients in these cohorts are presented in Table 7. The Finnish cohort consisted of 301 HLA-matched recipient/donor sibling pairs having clinical data and DNA samples sent for genotyping. The cohort also included 11 individual recipients and 8 donors without the respective sibling. All recipients underwent allogeneic HSCT at Helsinki University Hospital, Comprehensive Cancer Center, Stem Cell Transplantation Unit, Finland, between 1993 and 2006. The pairs were matched to low-resolution level at HLA-A, -B, and -DRB1 loci. HLA typing was performed with Lymphotype HLA-AB and Lymphotype HLA-DR-DQ (Bio-Rad Medical Diagnostics), LIPA Reverse Dot Blot (Innogenetics Group), or HLA-SSP (Pel Freez, Dynal Biotech LLC). The present cohort overlapped significantly with those utilized in our previous publications^6,12,21,26^. After genotyping and imputation, the cohort in the present study included 239 recipient/donor pairs, 23 individual recipients, and 28 individual donors. The majority (>75%) of GvHD prevention procedures combined cyclosporine, steroid, and 3 to 4 doses of methotrexate, while 18% received a combination of cyclosporine and mycophenolate mofetil.

**Table 7:** Characteristics of the Finnish and Spanish recipients.

Characteristic	Finnish recipients^*^	Spanish recipients^†^	*P*
Recipient age, median years (range)	49 (18–65)	50 (8–72)	0.085^‡^
Donor age, median years (range)	46 (4–65)	47 (3–78)	0.424^‡^
Recipient-donor gender, n (%)			
Male-male	73 (28)	87 (33)	0.479^§^
Male-female	57 (22)	62 (23)	
Female-female	61 (23)	51 (19)	
Female-male	71 (27)	66 (25)	
Diagnosis, n (%)			
Acute myeloid leukemia	73 (28)	88 (33)	0.203^§^
Acute lymphoblastic leukemia	39 (15)	24 (9)	0.036^§^
Chronic myeloid leukemia	37 (14)	13 (5)	<0.001^§^
Myelodysplastic syndrome	20 (8)	26 (10)	0.390^§^
Hodgkin’s lymphoma	0 (0)	12 (5)	0.001^§^
Non-Hodgkin’s lymphoma	12 (5)	50 (19)	<0.001^§^
Myeloma	56 (21)	38 (14)	0.032^§^
Aplastic anemia	4 (2)	5 (2)	1.000^||^
Other malignancies	21 (8)	11 (4)	0.060^§^
Stem cell source, n (%)			
Bone marrow	138 (53)	13 (5)	<0.001^§^
Peripheral blood	124 (47)	254 (95)	
Conditioning regimen, n (%)			
Myeloablative	199 (76)	110 (42)	<0.001^§^
Reduced intensity conditioning	63 (24)	151 (58)	
aGvDH grades III–IV, n (%)	23 (11)	39 (18)	0.044^§^
cGvHD, extensive, n (%)	71 (39)	54 (32)	0.156^§^

^a^GvHD indicates acute graft-versus-host disease; and cGvHD, chronic graft-versus-host disease.

*Finnish recipients underwent allogeneic HSCT at Helsinki University Hospital, Comprehensive Cancer Center, Stem Cell Transplantation Unit, Finland, between 1993 and 2006.

^†^Spanish recipient underwent allogeneic HSCT at 13 Spanish transplant centers between 2002 and 2014.

^‡^The significance of variation between characteristics in the study cohorts was analyzed using the non-parametric Mann–Whitney U-test.

^§^The significance of variation between characteristics in the study cohorts was analyzed using the Pearson chi-square test.

^||^The significance of variation between characteristics in the study cohorts was analyzed using the Fisher’s exact test.

The Spanish cohort was composed of 264 HLA-matched recipient/donor sibling pairs having clinical data and DNA samples sent for genotyping. The cohort also included 10 individual recipients and 30 donors without the respective sibling. HLA matching was completed at low-resolution at HLA-A and -B loci and at high-resolution at the HLA-DRB1 locus. Recipients received allogeneic HSCT between 2002 and 2014 at 13 Spanish transplant centers. After genotyping and imputation, the final study cohort was composed of 253 recipient/donor pairs, 15 individual recipients, and 30 individual donors. For GvHD prevention, 57% of recipients received a combination of cyclosporine and methotrexate, 10% received cyclosporine only, and 11% were treated with a combination of cyclosporine and mycophenolate mofetil.

The clinical outcomes examined were severe acute and chronic GvHD. The phenotypes compared were aGvHD grade 0 versus grades III–IV and absent cGvHD versus extensive cGvHD. Local determinations of GvHD grades were used. The samples were graded according to guidelines established by the European Society for Blood and Marrow guidelines^27,28^.

This study conformed to principles of the Declaration of Helsinki and was approved by the Ethics Committee of Helsinki University Central Hospital and the DNA bank of the Spanish Group for Stem Cell Transplantation (GETH). All participants gave written informed consent.

### Genotyping and imputation

Genotyping was performed at FIMM Technology Centre, Helsinki, Finland. DNA samples from the Finnish cohort were extracted using the QIAamp DNA Blood Mini Kit (Qiagen) from the white blood cell fraction of peripheral blood samples and sent for HLA typing. The Finnish cohort was genotyped using an Immunochip (Illumina) array comprising 196524 variants in 2013. DNA samples of the Spanish cohort were received from the DNA bank of the GETH. The array utilized for the analysis of Spanish samples from the years 2016–17 was the Infinium® ImmunoArray-24 v2.0 (Illumina), which comprises 253702 variants. Initial quality control identified samples with discordant sex information, duplicate samples, a call rate <97%, and heterozygosity excess <−0.3 (not X chromosome) or >0.2 and >0.1 for the X chromosome.

The autosomal genotype data were imputed with IMPUTE2 using 1000 Genomes Phase 3 as a phased reference panel^29^. Pre-filtering of the variants and samples was completed according to the methods described by Anderson *et al*.^30^. Individuals with a missing genotype >3%, variants with a minor allele frequency (MAF) <1%, variants with a missing data rate >5%, and variants with a HWE P-value < 1 × 10^−5^ were excluded. The principal components of both cohorts were determined and the imputation procedures were carried out separately. Post-imputation filtering excluded variants having an IMPUTE2 INFO-field measure of the observed statistical information <0.5^31^. After post-imputation filtering, 5041081 and 5737173 variants were included in the Finnish and Spanish cohort genotype datasets, respectively. The datasets analyzed during the current study are not publicly available due to limitations of ethical permits which do not allow distribution of personal data, including individual genetic and clinical results.

### Statistical analyses

The significance of variation between characteristics in the study cohorts was analyzed using the non-parametric Mann–Whitney U-test (recipient and donor age), Pearson’s chi-square test (transplant gender direction, diagnosis, stem cell source, condition regimen, and GvHD grade), or Fisher’s exact test (aplastic anemia diagnosis). P-values < 0.05 were considered statistically significant (Table 7).

Principal component analysis (PCA) was used to determine the genetic population structure of the two study cohorts. Non-imputed common SNPs shared by the two cohorts were included in the analysis. The SNPs were pruned to exclude strong linkage disequilibrium^32^. The analysis was executed with Plink v1.90b3u (www.cog-genomics.org/plink/1.9/)^33^ commands *indep-pairwise 50 5 0*.*8* and *pca*, and the result was plotted using R version 3.3.3^34^. A PCA-plot of the first two dimensions is presented in Supplementary Figure 1.

An association between the SNPs and acute and chronic GvHD was determined using the chi-square allelic test and is expressed as the OR with the 95% CI (Tables 1 and 2). The frequencies of the recipients and donors are presented in Supplementary Table 5. SNPs with a MAF < 0.01 and HWE 1 × 10^−5^ were excluded from the analysis. The current study evaluated results presented before and, therefore, despite multiple tests (77 in the Finnish and 97 in the Spanish cohorts), a P-value < 0.05 was considered to support a statistically significant replication. Statistical analyses were performed with IBM SPSS Statistics version 24 and PLINK 1.07^35^.

The eQTL analyses (Tables 3 and 4) were performed in February 2017 utilizing the comprehensive Blood eQTL Database (http://genenetwork.nl/bloodeqtlbrowser/) published by Westra *et al*.^16^. The database consists of both *cis*- and *trans*-eQTL results generated from a meta-analysis of seven studies including 5311 peripheral blood samples and a replication analysis with 2775 samples. Z-scores with a false detection rate (FDR) <0.05 were considered statistically significant.

The cytokine QTL database (https://hfgp.bbmri.nl/), recently published by Li Y *et al*.^17^, combines the host genetics and cytokine production after various microbial stimuli. The effects of candidate SNPs on the cytokine response were analyzed in March 2017 (Tables 5 and 6). P-values < 0.05 were considered to be statistically significant.

## Electronic supplementary material

Supplementary file

## References

[1] Blazar BR, Murphy WJ, Abedi M (2012). Advances in graft-versus-host disease biology and therapy. Nat. Rev. Immunol..

[2] Nassereddine S, Rafei H, Elbahesh E, Tabbara I (2017). Acute Graft Versus Host Disease: A Comprehensive Review. Anticancer Res..

[3] Ferrara JL, Levine JE, Reddy P, Holler E (2009). Graft-versus-host disease. Lancet.

[4] Warren EH (2012). Effect of MHC and non-MHC donor/recipient genetic disparity on the outcome of allogeneic HCT. Blood.

[5] Spierings E (2013). Multicenter Analyses Demonstrate Significant Clinical Effects of Minor Histocompatibility Antigens on GvHD and GvL after HLA-Matched Related and Unrelated Hematopoietic Stem Cell Transplantation. Biology of Blood and Marrow Transplantation.

[6] McCarroll SA (2009). Donor-recipient mismatch for common gene deletion polymorphisms in graft-versus-host disease. Nat. Genet..

[7] Penack O, Holler E, van den Brink MR (2010). Graft-versus-host disease: regulation by microbe-associated molecules and innate immune receptors. Blood.

[8] Elmaagacli AH (2011). Toll-like receptor 9, NOD2 and IL23R gene polymorphisms influenced outcome in AML patients transplanted from HLA-identical sibling donors. Bone Marrow Transplant..

[9] Harkensee C (2012). Single nucleotide polymorphisms and outcome risk in unrelated mismatched hematopoietic stem cell transplantation: an exploration study. Blood.

[10] Chien JW (2012). Evaluation of published single nucleotide polymorphisms associated with acute GVHD. Blood.

[11] Gam R (2017). Genetic Association of Hematopoietic Stem Cell Transplantation Outcome beyond Histocompatibility Genes. Frontiers in Immunology.

[12] Impola U (2014). Donor Haplotype B of NK KIR Receptor Reduces the Relapse Risk in HLA-Identical Sibling Hematopoietic Stem Cell Transplantation of AML Patients. Front. Immunol..

[13] Khaled SK (2016). Influence of Absorption, Distribution, Metabolism, and Excretion Genomic Variants on Tacrolimus/Sirolimus Blood Levels and Graft-versus-Host Disease after Allogeneic Hematopoietic Cell Transplantation. Biology of Blood and Marrow Transplantation.

[14] Byun JM (2016). The Impact of Methylenetetrahydrofolate Reductase C677T Polymorphism on Patients Undergoing Allogeneic Hematopoietic Stem Cell Transplantation with Methotrexate Prophylaxis. PLOS ONE.

[15] Hansen JA, Chien JW, Warren EH, Zhao LP, Martin PJ (2010). Defining genetic risk for graft-versus-host disease and mortality following allogeneic hematopoietic stem cell transplantation. Curr. Opin. Hematol..

[16] Westra HJ (2013). Systematic identification of trans eQTLs as putative drivers of known disease associations. Nat. Genet..

[17] Li Y (2016). A Functional Genomics Approach to Understand Variation in Cytokine Production in Humans. Cell.

[18] Lin MT (2003). Relation of an interleukin-10 promoter polymorphism to graft-versus-host disease and survival after hematopoietic-cell transplantation. N. Engl. J. Med..

[19] Jaskula E (2013). IL-10 promoter polymorphisms influence susceptibility to aGvHD and are associated with proportions of CD4+FoxP3+ lymphocytes in blood after hematopoietic stem cell transplantation. Tissue Antigens.

[20] Goussetis E (2011). Cytokine gene polymorphisms and graft-versus-host disease in children after matched sibling hematopoietic stem cell transplantation: a single-center experience. Cellular and Molecular Immunology.

[21] Sivula J, Turpeinen H, Volin L, Partanen J (2009). Association of IL-10 and IL-10Rβ gene polymorphisms with graft-versus-host disease after haematopoietic stem cell transplantation from an HLA-identical sibling donor. BMC Immunology.

[22] Petersdorf EW (2013). The major histocompatibility complex: a model for understanding graft-versus-host disease. Blood.

[23] Bari R (2015). Genome-wide single-nucleotide polymorphism analysis revealed SUFU suppression of acute graft-versus-host disease through downregulation of HLA-DR expression in recipient dendritic cells. Scientific Reports.

[24] Sato-Otsubo A (2015). Genome-wide surveillance of mismatched alleles for graft-versus-host disease in stem cell transplantation. Blood.

[25] Kim D (2012). Multiple Single-Nucleotide Polymorphism-Based Risk Model for Clinical Outcomes After Allogeneic Stem-Cell Transplantation, Especially for Acute Graft-Versus-Host Disease. Transplantation.

[26] Sivula J (2012). Toll-like receptor gene polymorphisms confer susceptibility to graft-versus-host disease in allogenic hematopoietic stem cell transplantation. Scand. J. Immunol..

[27] Przepiorka D (1995). 1994 Consensus Conference on Acute GVHD Grading. Bone Marrow Transplant..

[28] Shulman HM (1980). Chronic graft-versus-host syndrome in man. A long-term clinicopathologic study of 20 Seattle patients. Am. J. Med..

[29] Howie BN, Donnelly P, Marchini J (2009). A flexible and accurate genotype imputation method for the next generation of genome-wide association studies. PLoS Genet..

[30] Anderson CA (2010). Data quality control in genetic case-control association studies. Nat. Protocols.

[31] Marchini J, Howie B (2010). Genotype imputation for genome-wide association studies. Nat. Rev. Genet..

[32] Novembre J (2008). Genes mirror geography within Europe. Nature.

[33] Chang, C. C. *et al*. Second-generation PLINK: rising to the challenge of larger and richer datasets. *Gigascience* **4**, 7-015-0047-8. eCollection 2015 (2015). 10.1186/s13742-015-0047-8 PMC4342193 25722852

[34] R Development Core Team. R: A language and environment for statistical computing. R Foundation for Statistical Computing, Vienna, Austria (2008).

[35] Purcell S (2007). PLINK: a tool set for whole-genome association and population-based linkage analyses. Am. J. Hum. Genet..

