# Supplementary material for: Genetic polymorphism related to monocyte-macrophage function is associated with graft-versus-host disease
Source: Sci Rep. 2017 Nov 15;7:15666. doi: 10.1038/s41598-017-15915-3 (PMC5688060; doi:10.1038/s41598-017-15915-3)
Supplement: Supplementary file 1 — Supplementary file [file 41598_2017_15915_MOESM1_ESM.pdf]

Genetic polymorphism related to monocyte-macrophage function is associated with graft-versus-host disease

Kati Hyvärinen[1], Jarmo Ritari[1], Satu Koskela[1], Riitta Niittyvuopio[2], Anne Nihtinen[2], Liisa Volin[2], David Gallardo Giralt[3], and Jukka Partanen[1]

[1]Finnish Red Cross Blood Service, Helsinki, Finland; [2]Helsinki University Hospital, Comprehensive Cancer Center, Stem Cell Transplantation Unit, Helsinki, Finland; [3]Department of Hematology, Institut Català d'Oncologia, Hospital Dr. Josep Trueta, Girona, Spain

SUPPLEMENTARY DATA

Table S1. Analyzed SNPs in the Finnish cohort

Table S2. Analyzed SNPs in the Spanish cohort

Table S3. Summary of SNPs associated with GvHD in the Finnish cohort

Table S4. Summary of SNPs associated with GvHD in the Spanish cohort

Table S5. Recipient-donor frequencies in case/control association analysis

Figure S1 Legend

Figure S1. Principal component analysis of the study cohorts

References

Table S1. Analyzed SNPs in the Finnish cohort.

CHR	GENE annotation	Reference study	SNP*	POSITION GRCh37	A1	A2
1	MTHFR	Chien et al.[1]	rs1801131	11854476	G	T
1	IL23R	Chien et al.[1]	rs11209026	67705958	A	G
1	FCGR2A	Kim et al.[2]	rs1801274	161479745	G	A
1	IL10	Chien et al.[1]	rs1800872	206946407	T	G
1	IL10	Chien et al.[1]	rs1800871	206946634	A	G
1	IL10	Chien et al.[1]	rs1800896	206946897	C	T
2	IL1RI	Kim et al.[2]	rs3917225	102769302	A	G
2	IL1RI	Kim et al.[2]	rs2110726	102794282	A	G
2	IL1A	Noori-Daloii et al.[3]	rs1800587	113542960	A	G
2	IL1B	Resende et al.[4]	rs1143634	113590390	A	G
2	IL1B	Chien et al.[1]	rs16944	113594867	A	G
2	CTLA4	Harkensee et al.[5]	rs231775	204732714	A	G
2	CTLA4	Harkensee et al.[5]	rs231777	204733588	T	C
2	CTLA4	Chien et al.[1]	rs3087243	204738919	A	G
4	FLJ13197	Sivula et al.[6]	rs337629	38635358	A	G
4	FLJ13197	Sivula et al.[6]	rs4833079	38654681	C	T
4	IL2	Chien et al.[1],Harkensee et al.[5]	rs2069762	123377980	C	A
5	IL7RA	Shamim et al.[7]	rs1494558	35861068	T	C
5	IL7RA	Shamim et al.[7]	rs1494555	35871190	G	A
5	IL12B	Noori-Daloii et al.[3]	rs3212227	158742950	G	T
6	-	Petersdorf et al.[8]	rs209130	28867800	T	C
6	MICD	Petersdorf et al.[8]	rs2523957	29940260	G	A
6	TNF	Harkensee et al.[5], Chien et al.[1]	rs1799964	31542308	C	T
6	TNF	Chien et al.[1]	rs1800630	31542476	A	C
6	TNF	Takahashi et al.[23]	rs1800629	31543031	A	G
6	HSPA1L	Petersdorf et al.[8]	rs2075800	31777946	T	C
6	ATF6B/FKBPL	Petersdorf et al.[8]	rs3830076	32096244	T	C
6	NOTCH4	Petersdorf et al.[8]	rs394657	32187023	G	A
6	HLA-DOB	Petersdorf et al.[8]	rs2071479	32781112	T	C
6	HLA-DPB1	Petersdorf et al.[9]	rs9277534	33054807	G	A
6	HLA-DPB2	Sato-Otsubo et al.[10]	rs6937034	33079766	G	A
6	-	Petersdorf et al.[8]	rs107822	33175575	T	C

6	VEGFA	Chien et al.[1]	rs833061	43737486	T	C
6	IL17	Espinoza et al.[11]	rs2275913	52051033	A	G
7	IL6	Chien et al.[1]	rs1800795	22766645	G	C
10	FAS	Chien et al.[1], Harkensee et al.[5]	rs1800682	90749963	G	A
10	FAS	Kim et al.[2]	rs2234978	90771829	T	C
10	FAS	Kim et al.[2]	rs2862833	90775629	A	G
10	SUFU	Bari et al.[12]	rs17114803	104386934	C	T
10	SUFU	Bari et al.[12]	rs17114808	104391285	T	C
12	KRAS	Sato-Otsubo et al.[10]	rs1137282	25362777	G	A
12	IFNg	Harkensee et al.[5]	rs2069705	68555011	G	A
14	NFKBIA	Kim et al.[2]	rs2233409	35874270	A	G
16	VKORC1	Khaled et al.[13]	rs7294	31102321	T	C
16	NOD2	Harkensee et al.[5]	rs2111235	50733969	A	G
16	NOD2	Harkensee et al.[5]	rs6500328	50736656	G	A
16	NOD2	Jaskula et al.[14]	rs2066844	50745926	T	C
16	NOD2	Harkensee et al.[5]	rs17313265	50747704	T	C
17	RANTES/CCL5	Shin et al.[15]	rs2280788	34207405	C	G
17	-	Shin et al.[15]	rs2107538	34207780	T	C

CHR indicates chromosome; SNP, single nucleotide polymorphism; GRCh37, Genome Reference Consortium Human genome build 37; A1, minor allele; and A2, major allele.

*Reference SNP number

Table S2. Analyzed SNPs in the Spanish cohort.

CHR	GENE annotation	Reference study	SNP*	POSITION GRCh37	A1	A2
1	MTHFR	Chien et al.[1]	rs1801131	11854476	G	T
1	MTHFR	Byun et al.[16], Harkensee et al.[5]	rs1801133	11856378	A	G
1	TNFRII	Kim et al.[2]	rs945439	12248942	G	A
1	TNFRII	Chien et al.[1], Harkensee et al.[5]	rs1061622	12252955	G	T
1	IL23R	Elmaagacli et al.[22]	rs11209026	67705958	A	G
1	DARC	Sellami et al.[17]	rs2814778	159174683	C	T
1	FCGR2A	Kim et al.[2]	rs1801274	161479745	G	A
1	IL10	Chien et al.[1]	rs1800872	206946407	T	G
1	IL10	Chien et al.[1]	rs1800871	206946634	A	G
1	IL10	Chien et al.[1]	rs1800896	206946897	C	T
1	-	Sivula et al.[6]	rs2800230	225061429	A	G
1	-	Sivula et al.[6]	rs10737416	225074757	A	C
2	IL1RI	Kim et al.[2]	rs3917225	102769302	G	A
2	IL1RI	Kim et al.[2]	rs2110726	102794282	A	G
2	IL1A	Noori-Daloii et al.[3]	rs1800587	113542960	A	G
2	IL1B	Resende et al.[4]	rs1143634	113590390	A	G
2	IL1B	Chien et al.[1]	rs16944	113594867	A	G
2	CTLA4	Harkensee et al.[5]	rs231775	204732714	G	A
2	CTLA4	Harkensee et al.[5]	rs231777	204733588	T	C
2	CTLA4	Chien et al.[1]	rs3087243	204738919	G	A
3	TLR9	Xiao et al.[18]	rs352140	52256697	C	T
3	TLR9	Xiao et al.[18]	rs352139	52258372	T	C
4	FLJ13197	Sivula et al.[6]	rs337629	38635358	A	G
4	FLJ13197	Sivula et al.[6]	rs4833079	38654681	C	T
4	IL2	Chien et al.[1], Harkensee et al.[5]	rs2069762	123377980	C	A
5	IL7RA	Shamim et al.[7]	rs1494558	35861068	T	C
5	IL7RA	Shamim et al.[7]	rs1494555	35871190	G	A
5	IL12B	Noori-Daloii et al.[3]	rs3212227	158742950	G	T
6	-	Petersdorf et al.[8]	rs209130	28867800	C	T
6	MICD	Petersdorf et al.[8]	rs2523957	29940260	G	A
6	TNF	Harkensee et al.[5], Chien et al.[1]	rs1799964	31542308	C	T
6	TNF	Chien et al.[1]	rs1800630	31542476	A	C
6	TNF	Chien et al.[1]	rs1800629	31543031	A	G

6	HSPA1L	Petersdorf et al.[8]	rs2075800	31777946	T	C
6	ATF6B/FKBPL	Petersdorf et al.[8]	rs3830076	32096244	T	C
6	NOTCH4	Petersdorf et al.[8]	rs394657	32187023	G	A
6	HLA-DPB1	Petersdorf et al.[9]	rs9277534	33054807	G	A
6	HLA-DPB2	Sato-Otsubo et al.[10]	rs6937034	33079766	G	A
6	-	Petersdorf et al.[8]	rs107822	33175575	T	C
6	VEGFA	Chien et al.[1]	rs699947	43736389	A	C
6	VEGFA	Chien et al.[1]	rs833061	43737486	C	T
6	IL17	Espinoza et al.[11]	rs2275913	52051033	A	G
7	IL6	Chien et al.[1]	rs1800795	22766645	C	G
10	FAS	Chien et al.[1], Harkensee et al.[5]	rs1800682	90749963	G	A
10	FAS	Kim et al.[2]	rs2234978	90771829	T	C
10	FAS	Kim et al.[2]	rs2862833	90775629	G	A
10	SUFU	Bari et al.[12]	rs17114803	104386934	C	T
10	SUFU	Bari et al.[12]	rs17114808	104391285	T	C
12	KRAS	Sato-Otsubo et al.[10]	rs1137282	25362777	G	A
12	IFNg	Harkensee et al.[5]	rs2069705	68555011	G	A
13	BAFF	Clark et al.[19]	rs16972217	108935447	T	C
13	BAFF	Clark et al.[19]	rs7993590	108935902	T	A
13	BAFF	Clark et al.[19]	rs12428930	108939705	C	A
13	BAFF	Clark et al.[19]	rs2893321	108943034	G	A
14	NFKBIA	Kim et al.[2]	rs2233409	35874270	A	G
16	IL4RA	Noori-Daloii et al.[3]	rs1801275	27374400	G	A
16	VKORC1	Khaled et al.[13]	rs7294	31102321	T	C
16	NOD2	Harkensee et al.[5]	rs2111235	50733969	A	G
16	NOD2	Harkensee et al.[5]	rs6500328	50736656	G	A
16	NOD2	Jaskula et al.[14]	rs2066844	50745926	T	C
16	NOD2	Harkensee et al.[5]	rs17313265	50747704	T	C
16	NOD2	Elmaagacli et al.[20]	rs2066847	50763778	I	D
19	TGFB1	Kim et al.[2]	rs1800469	41860296	A	G
21	IL10RB	Lin et al.[21]	rs2834167	34640788	G	A

Abbreviations are explained in Table S2.

*Reference SNP number

Table S3. Summary of SNPs associated with GvHD in the Finnish cohort.

Gene, location*	SNP	Reference study	Risk/ protective†	Genotyped/ Imputed	CHR	A1	A2	MAF	HWE P-value	Call rate	IMPUTE2 INFO metric‡
IL1B, upstream variant 2KB	rs16944	Harkensee et al.[5]	Patient AA protective	Imputed	2	A	G	0.383	0.653	0.98	0.990
IL1R1, intron	rs3917225	Kim et al.[2]	Donor GG risk	Genotyped	2	A	G	0.375	0.588	1	1
MICD,-	rs2523957	Petersdorf et al.[8]	Patient/donor mismatch risk	Genotyped	6	G	A	0.318	0.001	0.99	1
TNF, upstream variant 2KB	rs1800629	Takahashi et al.[23]	Donor A risk	Genotyped	6	A	G	0.110	0.664	1	1
NFKBIA, upstream variant 2KB	rs2233409	Kim et al.[2]	Recipient T protective	Genotyped	14	A	G	0.200	1	1	1
NOD2, intron	rs6500328	Harkensee et al.[5]	Donor GG protective	Genotyped	16	G	A	0.446	0.864	1	1
HSPA1L, missense	rs2075800	Petersdorf et al.[8]	Patient AA protective	Genotyped	6	T	C	0.293	0.470	0.97	1
PRSS53, upstream variant 2KB/ VKRC1, utr variant 3 prime	rs7294	Khaled et al.[13]	Patient AA risk	Imputed	16	T	C	0.407	0.378	0.99	0.991
KRAS, synonymous codon	rs1137282	Sato-Otsubo et al.[10]	Patient/donor mismatch risk	Imputed	12	G	A	0.144	0.380	0.99	0.939

SNP indicates single nucleotide polymorphism; CHR, chromosome; A1, minor allele; A2, major allele; MAF, minor allele frequency; and HWE, Hardy–Weinberg equilibrium.

*Annotation of SNP according to National Center for Biotechnology Information dbSNP database.

† Risk/protective outcome status of the SNP in the reference study.

‡Measure of the observed statistical information associated with the allele frequency estimate.

Table S4. Summary of SNPs associated with GvHD in the Spanish cohort.

Gene, location*	SNP	Reference study	Risk/ protective†	Genotyped/ Imputed	CHR	A1	A2	MAF	HWE P-value	Call rate	IMPUTE2 INFO metric
Not in gene	rs2800230	Sivula et al.[6]	Donor CT risk	Genotyped	1	A	G	0.462	0.050	1	1
IL1A, utr variant 5 prime	rs1800587	Noori-Daloii et al.[3]	Donor CC risk	Genotyped	2	A	G	0.290	0.758	1	1
IL1B, synonymous codon	rs1143634	Resende et al.[4]	Donor T protective	Genotyped	2	A	G	0.231	0.339	1	1
IL10, upstream variant 2KB	rs1800872	Harkensee et al.; Chien et al.[1, 5]	Donor haplotype CCG risk[5]; Recipient T protective[1]	Imputed	1	T	G	0.267	0.233	1	0.996
IL10, upstream variant 2KB	rs1800871	Harkensee et al.; Chien et al.[1, 5]	Donor haplotype CCG risk[5]; Recipient A protective[1]	Genotyped	1	A	G	0.267	0.233	1	1
IL10, upstream variant 2KB	rs1800896	Harkensee et al.[5]	Donor AA protective	Genotyped	1	C	T	0.441	0.302	1	1
IL10RB, missense	rs2834167	Lin et al.[21]	Recipient A and donor G protective	Genotyped	21	G	A	0.251	0.500	1	1
IL23R, missense	rs11209026	Elmaagacli et al.[22]	Donor A protective	Genotyped	1	A	G	0.058	0.416	1	1
LOC105373109, intron	rs10737416	Sivula et al.[6]	Patient AA protective	Imputed	1	A	C	0.401	0.647	0.93	0.942
TLR9, synonymous codon	rs352140	Xiao et al.[18]	Donor C risk	Imputed	3	C	T	0.482	0.727	0.94	0.955
TLR9, intron	rs352139	Xiao et al.[18]	Donor A risk	Genotyped	3	T	C	0.458	0.443	1	1
FAS, downstream variant 500B	rs2862833	Kim et al.[2]	Recipient C risk	Genotyped	10	G	A	0.476	0.800	1	1

Abbreviations are explained in Table S4.

*Annotation of SNP according to National Center for Biotechnology Information dbSNP database.

† Risk/protective outcome status of the SNP in the reference study.

‡Measure of the observed statistical information associated with the allele frequency estimate.

Table S5. Recipient-donor frequencies in case/control association analysis.

	Finnish cohort		Spanish cohort	
	**Cases**	**Controls**	**Cases**	**Controls**
**Recipients**				
aGvHD	23	180	37	170
cGvHD	71	112	56	115
**Donors**				
aGvHD	28	188	41	183
cGvHD	73	118	58	120

The association was determined with the 1 df chi-square allelic test case/control association analysis and expressed as odds ratios with 95% confidence interval**.**

aGvHD indicates acute graft-versus-host disease grade III–IV; and cGvHD, extensive chronic graft-versus-host disease.

Figure S1 Legend.

Principal component analysis -based genetic clustering of the study cohorts. The Finnish and Spanish cohorts are indicated by blue and red colour, respectively. The analysis is based on non-imputed and LD-pruned SNPs shared by the two populations. Each data point represents an individual sample. The percent of variance explained by the two main components is indicated in parentheses.

Figure S1. Principal component analysis of the study cohorts

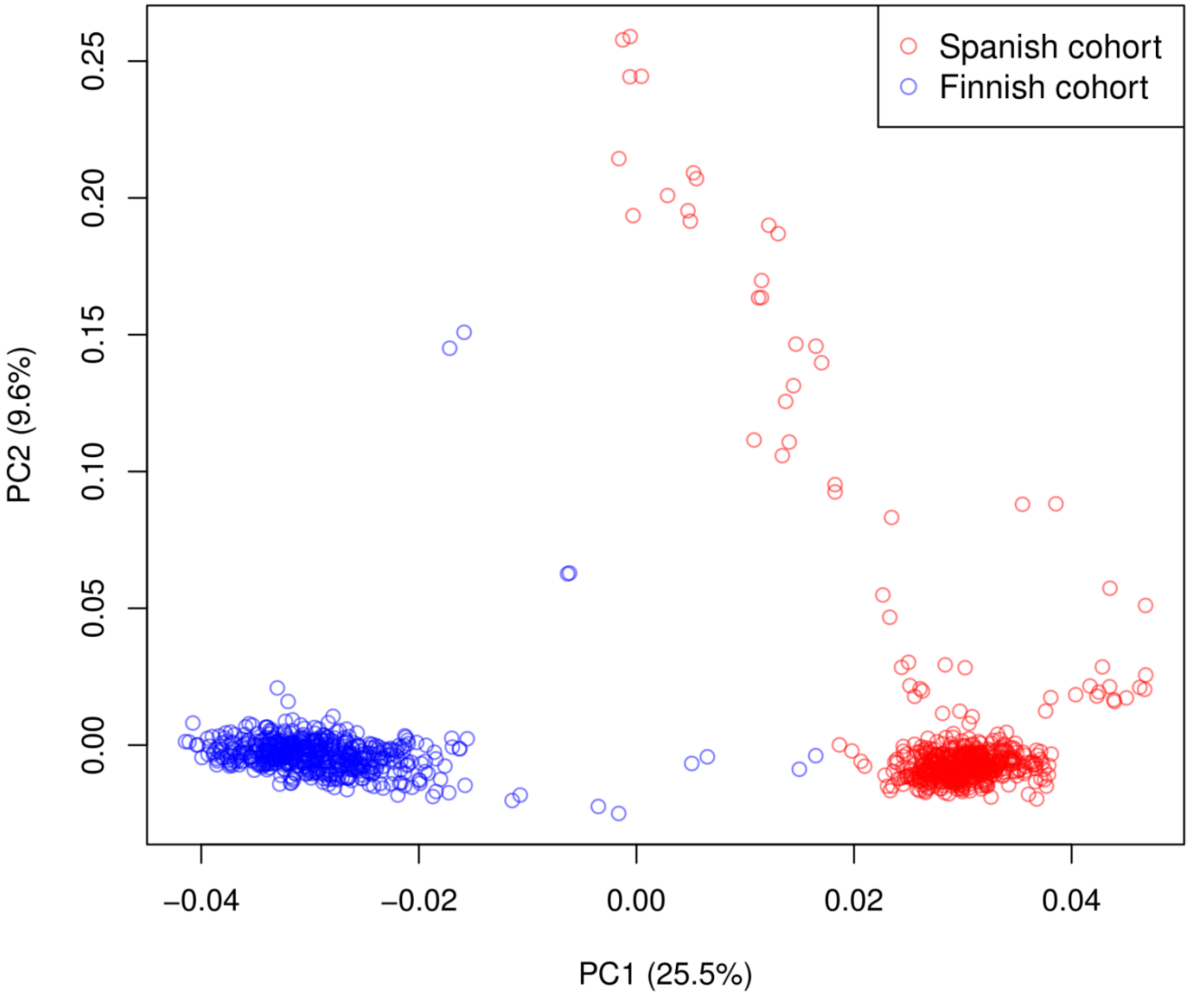

References

1. Chien, J. W. *et al*. Evaluation of published single nucleotide polymorphisms associated with acute GVHD. *Blood* **119**, 5311-5319 (2012).

2. Kim, D. *et al*. Multiple Single-Nucleotide Polymorphism-Based Risk Model for Clinical Outcomes After Allogeneic Stem-Cell Transplantation, Especially for Acute Graft-Versus-Host Disease. *Transplantation* **94**, 1250-1257 (2012).

3. Noori-Daloii, M. *et al*. Cytokine gene polymorphism and graft-versus-host disease: a survey in Iranian bone marrow transplanted patients. *Mol. Biol. Rep.* **40**, 4861-4867 (2013).

4. Resende, R. *et al*. Association Between IL1B (+3954) Polymorphisms and IL-1β Levels in Blood and Saliva, Together with Acute Graft-Versus-Host Disease. *Journal of Interferon & Cytokine Research* **33**, 392-397 (2013).

5. Harkensee, C. *et al*. Single nucleotide polymorphisms and outcome risk in unrelated mismatched hematopoietic stem cell transplantation: an exploration study. *Blood* **119**, 6365-6372 (2012).

6. Sivula, J. *et al*. Toll-like receptor gene polymorphisms confer susceptibility to graft-versus-host disease in allogenic hematopoietic stem cell transplantation. *Scand. J. Immunol.* **76**, 336-341 (2012).

7. Shamim, Z. *et al*. Polymorphism in the interleukin-7 receptor-alpha and outcome after allogeneic hematopoietic cell transplantation with matched unrelated donor. *Scand. J. Immunol.* **78**, 214-220 (2013).

8. Petersdorf, E. W. *et al*. Mapping MHC haplotype effects in unrelated donor hematopoietic cell transplantation. *Blood* **121**, 1896-1905 (2013).

9. Petersdorf, E. W. *et al*. High HLA-DP Expression and Graft-versus-Host Disease. *N. Engl. J. Med.* **373**, 599-609 (2015).

10. Sato-Otsubo, A. *et al*. Genome-wide surveillance of mismatched alleles for graft-versus-host disease in stem cell transplantation. *Blood* **126**, 2752-2763 (2015).

11. Espinoza, J. L. *et al*. A genetic variant in the IL-17 promoter is functionally associated with acute graft-versus-host disease after unrelated bone marrow transplantation. *PLoS One* **6**, e26229 (2011).

12. Bari, R. *et al*. Genome-wide single-nucleotide polymorphism analysis revealed SUFU suppression of acute graft-versus-host disease through downregulation of HLA-DR expression in recipient dendritic cells. *Scientific Reports* **5**, 11098 (2015).

13. Khaled, S. K. *et al*. Influence of Absorption, Distribution, Metabolism, and Excretion Genomic Variants on Tacrolimus/Sirolimus Blood Levels and Graft-versus-Host Disease after Allogeneic Hematopoietic Cell Transplantation. *Biology of Blood and Marrow Transplantation* **22**, 268-276 (2016).

14. Jaskula, E. *et al*. NOD2/CARD15 Single Nucleotide Polymorphism 13 (3020insC) is Associated with Risk of Sepsis and Single Nucleotide Polymorphism 8 (2104C>T) with Herpes Viruses Reactivation in Patients after Allogeneic Hematopoietic Stem Cell Transplantation. *Biology of Blood and Marrow Transplantation* **20**, 409-414 (2014).

15. Shin, D. *et al*. RANTES Polymorphisms and the Risk of Graft-versus-Host Disease in Human Leukocyte Antigen-Matched Sibling Allogeneic Hematopoietic Stem Cell Transplantation. *Acta Haematol.* **129**, 137-145 (2013).

16. Byun, J. M. *et al*. The Impact of Methylenetetrahydrofolate Reductase C677T Polymorphism on Patients Undergoing Allogeneic Hematopoietic Stem Cell Transplantation with Methotrexate Prophylaxis. *PLOS ONE* **11**, e0163998 (2016).

17. Sellami, M. H. *et al*. Evidence that erythrocyte DARC-positive phenotype can affect the GVHD occurrence after HLA-identical sibling HSCT. *Transpl. Immunol.* **25**, 148-152 (2011).

18. Xiao, H. W. *et al*. Donor TLR9 gene tagSNPs influence susceptibility to aGVHD and CMV reactivation in the allo-HSCT setting without polymorphisms in the TLR4 and NOD2 genes. *Bone Marrow Transplant.* **49**, 241-247 (2014).

19. Clark, W. B. *et al*. Genetic variation in recipient B-cell activating factor modulates phenotype of GVHD. *Blood* **118**, 1140-1144 (2011).

20. Elmaagacli, A. H. *et al*. Toll-like receptor 9, NOD2 and IL23R gene polymorphisms influenced outcome in AML patients transplanted from HLA-identical sibling donors. *Bone Marrow Transplant.* **46**, 702-708 (2011).

21. Lin, M.T. *et al*. Genetic variation in the IL-10 pathway modulates severity of acute graft-versus-host disease following hematopoietic cell transplantation: synergism between IL-10 genotype of patient and IL-10 receptor β genotype of donor. *Blood* **106**, 3995-4001 (2005).

22. Elmaagacli, A. H. *et al*. Relation of an interleukin-23 receptor gene polymorphism to graft-versus-host disease after hematopoietic-cell transplantation. *Bone Marrow Transplant.* **41**, 821-826 (2008).

23. Takahashi, H. *et al.* Contribution of TNF-alpha and IL-10 gene polymorphisms to graft-versus-host disease following allo-hematopoietic stem cell transplantation. *Bone Marrow Transplant.* **26**, 1317-1323 (2000).
